# CT Myocardial Perfusion Imaging: A New Frontier in Cardiac Imaging

**DOI:** 10.1155/2018/7295460

**Published:** 2018-10-14

**Authors:** Sara Seitun, Cecilia De Lorenzi, Filippo Cademartiri, Angelo Buscaglia, Nicole Travaglio, Manrico Balbi, Gian Paolo Bezante

**Affiliations:** ^1^Department of Radiology, Policlinico San Martino Hospital, Genoa, Italy; ^2^Cardiovascular Imaging Center, SDN IRCCS, Naples, Italy; ^3^Clinic of Cardiovascular Diseases, Policlinico San Martino Hospital, Genoa, Italy

## Abstract

The past two decades have witnessed rapid and remarkable technical improvement of multidetector computed tomography (CT) in both image quality and diagnostic accuracy. These improvements include higher temporal resolution, high-definition and wider detectors, the introduction of dual-source and dual-energy scanners, and advanced postprocessing. Current new generation multidetector row (≥64 slices) CT systems allow an accurate and reliable assessment of both coronary epicardial stenosis and myocardial CT perfusion (CTP) imaging at rest and during pharmacologic stress in the same examination. This novel application makes CT the unique noninvasive “one-stop-shop” method for a comprehensive assessment of both anatomical coronary atherosclerosis and its physiological consequences. Myocardial CTP imaging can be performed with different approaches such as static arterial first-pass imaging, and dynamic CTP imaging, with their own advantages and disadvantages. Static CTP can be performed using single-energy or dual-energy CT, employing qualitative or semiquantitative analysis. In addition, dynamic CTP can obtain quantitative data of myocardial blood flow and coronary flow reserve. The purpose of this review was to summarize all available evidence about the emerging role of myocardial CTP to identify ischemia-associated lesions, focusing on technical considerations, clinical applications, strengths, limitations, and the more promising future fields of interest in the broad spectra of ischemic heart disease.

## 1. Introduction

Since the clinical introduction of multidetector computed tomography (CT) in the late 1990s [1, 2], coronary computed tomography angiography (CTA) has become the mainly used noninvasive imaging modality in the suspicion of coronary artery disease (CAD).

In consideration of its high sensitivity and negative predictive value (≥95%) for the detection of significant coronary stenosis [3], coronary CTA is currently recommended as the first diagnostic test in symptomatic, low-to-intermediate risk population [4–6].

The EVINCI (EValuation of INtegrated Cardiac Imaging for the Detection and Characterization of Ischaemic Heart Disease) study, a prospective multicenter European comparative effectiveness trial, has demonstrated that comprehensive assessment of anatomic CAD by CTA had a sensitivity of 91% and specificity of 92%, which were higher than functional test including myocardial perfusion imaging by either single photon emission computed tomography (SPECT) or positron emission tomography (PET) and ventricular wall motion imaging by either stress echocardiography or magnetic resonance imaging (MRI) [7]. However, this study has several limitations that may explain the lower accuracy of functional imaging, such as the lack of additional information (myocardial perfusion analysis and late-gadolinium enhancement for MRI and quantitative myocardial perfusion for PET), and the submaximal stress for echocardiography in 41% of cases [7].

The effects on clinical decision-making due to incorporation of CTA in the chest pain care pathway, jointly with its safety, are demonstrated in the SCOT-HEART [8] and in the PROMISE [9] prospective multicenter trials.

Coronary CTA has also a prognostic value providing information on the total plaque burden, with a better outcome when there is no evidence of CAD and a worse prognosis in case of detection of coronary atherosclerosis, depending on its severity and extension [10–15].

Moreover, it has the ability to detect nonobstructive non-flow-limiting CAD, helping to identify patients at risk of future cardiac events with more precision than functional testing [16].

The actual limit of coronary CTA is the impossibility to assess the functional significance of coronary stenosis related to its moderate positive predictive value (about 50%) in detecting inducible myocardial ischemia [17–19].

Physiological evaluation needs to be improved, because it influences the outcome of CAD more than its anatomical detection. In fact, studies using fractional flow reserve (FFR) have demonstrated that ischemia-guided coronary revascularization, especially percutaneous coronary intervention (PCI), is superior to angiography-guided strategy [20–23].

In absence of myocardial ischemia, revascularization is associated with no symptomatic or prognostic benefit for patients, while it is effective in patients with moderate to severe ischemia (total myocardial ischemia >10%) [20].

The evidence of coronary stenosis, especially for luminal narrowing of 30-70%, is not predictive of inducible ischemia. Further functional test, such as SPECT, stress echocardiography, or stress perfusion MRI are needed to guide revascularization indication [19].

Furthermore, a subanalysis of the EVINCI study has shown that, in patients at intermediate risk of CAD, hybrid imaging with CTA and SPECT allows noninvasive colocalization of myocardial perfusion defects and subtending coronary arteries, impacting clinical decision-making in almost one in every five subjects [24].

In the last decades, we observed a rapid technological improvement, which led to a notable reduction of the scan time, motion artifacts, use of contrast agent, and radiation dose exposure, while yielding, at the same time, higher spatial and temporal resolution [25–27] which widened the application of CT from anatomical detection of CAD to physiological assessment of myocardial ischemia leading to the first human report of stress myocardial CT perfusion (CTP) by Kurata et al. in 2005 [28]. Currently, feasibility of CTP imaging with modern multidetector row (≥64 slices) CT systems at rest and during pharmacologic stress [29–35] has been demonstrated by several clinical studies and recent multicenter trials.

The purpose of this review was to summarize all available evidence about the emerging role of myocardial CTP in the assessment of the hemodynamic impact of coronary lesions, focusing on technical considerations, clinical applications, strengths, limitations, and the more promising future field of interest in the broad spectra of ischemic heart disease.

## 2. The Physiologic Basis of Stress Myocardial Perfusion

In the classic ischemic cascade, perfusion abnormalities are the first to occur, before metabolic alterations, wall motion abnormalities, ECG changes, and symptoms.

Stress tests evaluating myocardial perfusion have a higher sensitivity in detecting flow-limiting stenosis compared with other imaging modalities based on the induction of stress-induced wall motion abnormalities or ECG changes [19].

Gould in 1974 was the first to investigate the relationship between luminal artery narrowing and the maximal hyperemic response [36].

Thanks to coronary autoregulation, involving myogenic and metabolic mechanism, myocardial perfusion at rest is normal until the luminal diameter narrowing of a coronary artery exceeds 85-90%.

However, in presence of coronary stenosis greater than 45% maximal coronary hyperemia induced by coronary arteriolar vasodilator leads to a progressive decrease in the hyperemic response [36].

In this situation, exercise or pharmacological vasodilation of subepicardial resistance vessels results in a reduction in distal coronary pressure that redistributes flow away from the subendocardium, leading to a “transmural steal” phenomenon [19].

Pharmacological stress agents are used to induce the hyperemic response in patients who cannot afford exercise test, that is, the preferred method to induce myocardial hyperemia.

For stress CTP, the most used substances are adenosine and dipyridamole that lead to arteriolar vasodilation by both direct and endothelium-mediated mechanisms through stimulation of A2A receptors in the microvasculature. In the absence of microcirculatory dysfunction, the vasodilatory response is associated with a 3.5- to 4-fold increase in myocardial blood flow [34].

Two intravenous (IV) lines are essential in CTP imaging for injection of the contrast media and of the vasodilator agent, respectively.

Adenosine is a powerful, endogenous molecule with a nonselective activation of four distinct subtypes (A1, A2A, A2B, and A3) receptors. Its infusion rate is 140 mcg/kg/min for 3 to 5 minutes with an infusion pump. Side effects could be AV block, peripheral vasodilation, and bronchospasm, but the most common are flushing, chest pain, dyspnea, dizziness, or nausea. Compared to dipyridamole, adenosine has a more rapid onset of action and a shorter half-life of 30 s; therefore most side effects resolve in a few seconds after discontinuation of the adenosine infusion.

Dipyridamole increase intracellular and interstitial concentration of adenosine, indirectly leading to coronary arteriolar vasodilatation, and it does not require an IV pump for infusion as it can be applied manually at a slow rate in a dose of 0.56 mg/kg to 0.84 mg/kg over a 4- to 6-minute period. Due to its longer half-life of approximately 30 minutes, dipyridamole-stress patients may require administration of aminophylline (slow intravenous injection of 50 mg to 250 mg) for reversal of persistent symptoms [19, 34, 37].

Recently, a new agent named regadenoson, an A2A selective agonist administered via a prefilled syringe in a single dose (400 mg) over 10 seconds, has been introduced as a pharmacologic stress vasodilator. It has a safer side effect profile in comparison to adenosine and dipyridamole, especially for patients with asthma or severe chronic obstructive pulmonary disease but it is limited by its cost and it is not widely available.

Regadenoson has been shown to be accurate for the detection of obstructive CAD in nuclear perfusion imaging, stress echocardiography, and, more recently, stress CTP studies [29, 38], even if a recent study by Johnson and Gould using quantitative Rb-82 PET imaging [38] showed a lower vasodilatory effect of regadenoson stress compared to dipyridamole stress, with an efficacy around 80%.

Of note, the myocardial perfusion can be evaluated by dobutamine as well [31]. The synthetic catecholamine dobutamine is primarily a *β*1-adrenergic receptors agonist, with mild effect on *α*1- and *β*2-receptors [39]. At low doses (≤ 10 *μ*g/kg/min), dobutamine improves myocardial contractility and induces coronary vasodilation; at higher doses (20-40 *μ*g/kg/min), it causes systematic vasodilation and serves as a positive chronotrope [39]. At these high doses, dobutamine mainly acts through increased of heart rate and myocardial oxygen consumption rather than “steal phenomenon” [31]. In the clinical practice, dobutamine stress is widely accepted as a noninvasive tool for stress echocardiography or stress MRI to detect myocardial ischemia by identifying regional wall motion abnormalities (RWMA), with similar accuracy and sensitivity of dipyridamole-stress imaging [39, 40]. Contrast-enhanced echocardiography and perfusion MRI may further improve diagnostic accuracy of dobutamine stress in detecting myocardial ischemia [39, 40].

Dobutamine is not the preferred pharmacological stressor in CTP imaging. However, as described by a recent case report, it may have a value to risk stratify patients with an anomalous coronary artery, since vasodilator stress imaging might not be sufficiently sensitive to identify dynamic coronary compression [41].

## 3. Protocol of CTP Imaging

The protocol of CTP imaging includes evaluation of myocardial perfusion during both rest (baseline) and stress (hyperemia) conditions and it is similar to other noninvasive imaging techniques such as stress cardiac MRI and nuclear imaging [19].

CTP analysis is performed after administration of iodinated contrast through an antecubital IV access by imaging the left ventricular (LV) myocardium during the first pass of the contrast bolus. Iodinated contrast attenuates X-rays directly proportionally to iodine content in tissue; thus myocardial perfusion defects can be directly visualized as hypoattenuated or nonenhancing regions.

Imaging during the early portion of first-pass circulation is critical, since after about 1 min a rapid wash-out of contrast agent due to diffusion to the extravascular space is expected [34].

Contrast injection needs, at high flow rate, at least 5 mL/s for optimizing the strength of enhancement in the first-pass arterial phase [34].

There are two protocols mostly used, named according to sequence of scan acquisitions: rest/stress or stress/rest. An interval of 10-15 minutes between the two sequences provides optimal contrast wash-out [19, 34].

The rest/stress protocol uses the ability of coronary CTA to rule out obstructive CAD. CTP is performed only in the presence of anatomically defined CAD of intermediate or obstructive degree, avoiding further radiation and iodinated contrast exposure in absence of coronary artery stenosis. This protocol is limited by the cross-contamination of contrast in the stress phase and beta-blocker administrated before the rest acquisition, leading to underestimation of myocardial ischemia.

The stress/rest protocol avoiding the risk of residual contrast media derived from the rest phase that may confound perfusion defects is optimized for the detection of myocardial ischemia.

The contrast media contamination of the rest phase may decrease sensitivity for infarction [19, 34].

Definitely, the best approach should be tailored on the patient's risk profile, reserving the rest/stress CTP for patients with low-to-intermediate pretest probability of CAD and stress/rest CTP for patients with high pretest probability of ischemia-associated lesions [34].

Myocardial CTP imaging can be performed with different approaches such as static arterial first-pass imaging and dynamic time-resolved CTP imaging, with their own advantages and disadvantages.

### 3.1. Static CTP Imaging: Monoenergetic CT Acquisition

The static CTP imaging is based on acquisition of one single phase during the first-pass of the contrast agent. Certain technical challenges involving scan timing relative to maximum contrast enhancement and optimal contrast material delivery must be met [19].

Generally, rest myocardial CTP imaging is derived from the coronary CTA examination.

ECG-gating of coronary CTA or CTP can be retrospective (with prospective tube current modulation) [42, 43] but also prospective, which is a new feature of latest multislice CT scanners (64 or more slices), allowing a significant reduction in radiation dose (less than 5 mSv), without causing any significant decrease in image quality [44, 45].

Prospectively ECG-triggered high-pitch spiral acquisition implemented with the second-generation 128-slice dual-source CT (DSCT) scanner allows the acquisition of the volumetric data of the heart in a single cardiac cycle with radiation exposure as low as 1 mSv [27, 46].

Visual qualitative assessment is the analyzing method of static CTP. Thick multiplanar reconstructions of approximately 5 mm to 8 mm are usually recommended for myocardial perfusion analysis to improve the contrast-to-noise ratio.

Myocardial contrast enhancement increases proportionally with iodine concentration, so perfusion defects appear as hypodense region with subendocardial or transmural distribution with respect to the normal myocardium.

An integrated review of stress and rest images is important to characterize not only ischemic from nonischemic myocardium, but also viable versus nonviable myocardium by differentiating between fixed and inducible perfusion abnormalities [19].

Hypoperfusion in stress with normal perfusion in rest underlines ischemia, whereas hypoperfusion in stress that persists with same extension in rest is indicative of necrosis [47]. Furthermore, hypoperfusion in stress that persists in rest with less extension than in stress is specific for peri-infarct ischemia [47]. A relative hyperenhancement to differing degrees of an infarct may be visualized on the second sequence of the acquisition protocol due to contrast distribution into the extravascular, extracellular interstitial space [19, 47].

**Figure 1:**
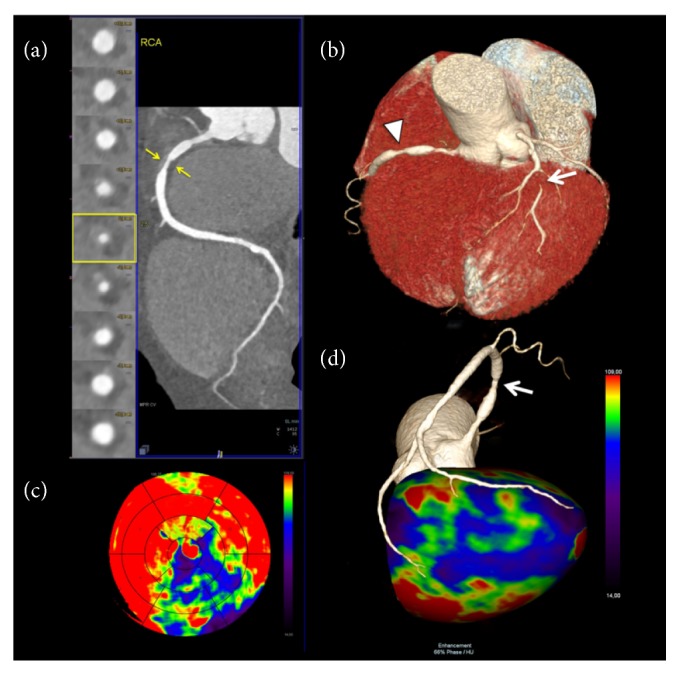
**Static single-energy CTP imaging**. 61-year-old male patient with multiple cardiovascular risk factors (smoke, hypercholesterolemia, and hypertension) presented with recurrent atypical chest pain. (a) Coronary CT angiography curved multiplanar reconstruction of the right coronary artery (RCA) with the corresponding orthogonal views showed a critical stenosis (>70% luminal narrowing) at the proximal segment sustained by a large noncalcified atherosclerotic plaque with positive remodeling (arrows). (b) Three-dimensional volume-rendering reconstruction demonstrating the critical stenosis of the proximal RCA (arrowhead) and showing also a tight stenosis (>90%, arrow) of the main diagonal branch (arrow). (c) 17-segment polar plot display of CT perfusion data acquired with a prospectively ECG-triggered high-pitch spiral technique at stress during the first pass, arterial phase, showed large, and severe perfusion defect color-coded in violet/blue/green at the inferior and inferolateral wall; note also a severe area of hypoperfusion in the apical lateral segment and in the apex. (d) Three-dimensional volume-rendering modeling of the left ventricular myocardial perfusion data with superimposed coronary tree (inferior view) showed the critical stenosis of the proximal segment of RCA (arrow) associated with an extensive perfusion defect at the inferior and inferolateral wall extending to apex (color-coded in violet/blue/green).

The final step to the analysis of a CTA/CTP study is the match of perfusion defects with the anatomic localization of coronary epicardial stenosis, Figure 1. This is crucial for the interpretation of the hemodynamic significance of CAD [19, 34].

Automated software application provides analysis of semiquantitative metrics such as the transmural perfusion ratio (TPR), determined as the ratio of subendocardial to mean subepicardial contrast attenuation, which has been already initially validated for MRI perfusion. However, the accuracy of TPR may be significantly affected by motion and beam-hardening artifacts or by a thinning myocardial wall in the context of prior infarction [47].

In conclusion, the patient specific ischemic burden may be determined in terms of volume of CT perfusion defect or percentage of ischemic myocardium relative to global myocardial volume [48].

### 3.2. Static CTP Imaging: Dual-Energy CT Acquisition

Dual-energy CT (DECT) myocardial perfusion imaging technology provides additional information about myocardial tissue composition compared with conventional single-energy computed tomography (SECT). Moreover, DECT improves limitations that are commonly present in SECT such as beam-hardening artifacts and blooming artifacts by using monochromatic image reconstruction [49].

Based on the specific attenuation spectral characteristics of the different tissues when exposed to two different photon energy levels, DECT enables distinguishing the features of the tissue and evaluating the myocardial blood supply by mapping iodine distribution within the myocardium [50].

**Figure 2:**
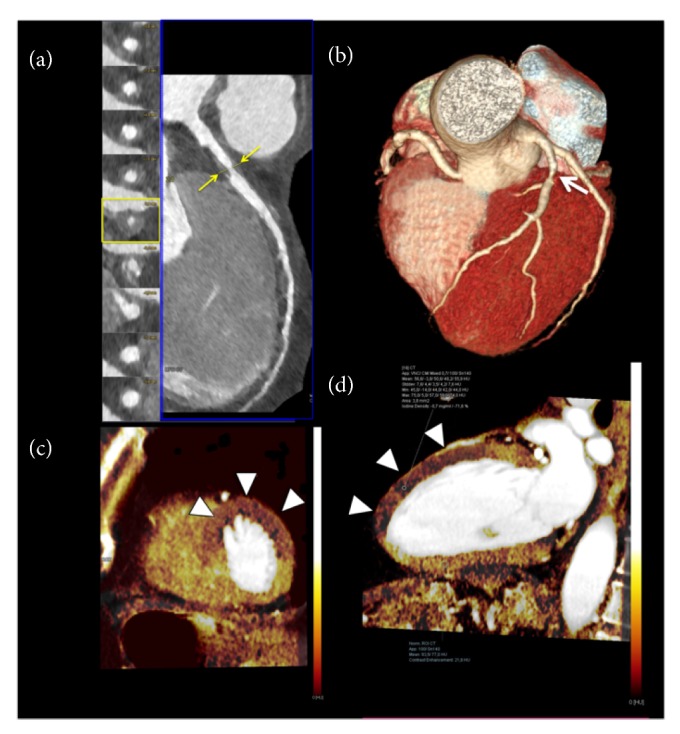
**Static retrospectively ECG gated dual-energy myocardial perfusion imaging**. 56-year-old man with multiple cardiovascular risk factors and stable angina. (a) Coronary CT angiography curved multiplanar reconstruction of the left anterior descending artery (LAD) with the corresponding perpendicular views showed a critical stenosis (>70% luminal narrowing) at the proximal segment (arrows) sustained by a large concentric predominately noncalcified plaque with positive remodeling (Remodeling Index= 2.1). (b) The corresponding three-dimensional volume-rendering reconstruction demonstrating the critical stenosis of the proximal LAD (arrow). (c-d) Myocardial short-axis (c) and 2-chamber long-axis (d) color-coded iodine distribution maps of dual-energy CTP imaging during stress showed perfusion defects at the antero-septal, anterior, and antero-lateral wall corresponding to the territory of the left anterior descending artery (arrowheads). Quantitative analysis of the dual-energy map at the level of the anterior wall shows a 71.6% reduction in iodine content (Iodine Density: −0.7 mg/ml) with respect to the remote myocardium at the inferior wall.

Iodine map provides a measure of per-voxel iodine myocardial concentration expressed in mg/mL, which improves accuracy when compared to standard visual analysis [34, 49, 51], Figure 2.

Different vendor-specific CT technologies have been developed to perform dual-energy acquisitions. Dual X-ray source system (Siemens Healthcare) is the most commonly used technology: there are two independent tubes paired with two detectors that simultaneously emit high (140-150 kV) and low (80-90-100 kV) energy levels [52].

A second modality is based on single-source CT with rapid (about 0.25 ms) switching of tube voltage between 80 and 140 kV either in a single gantry rotation (GSI Cardiac, GE Healthcare) or in sequential rotations (Acquilion One, Toshiba) [19].

The dual-layer (“sandwich”) detector (Philips Healthcare) is an alternative approach made of two different materials able to differentiate between low and high energy photons, with the source operating at constant tube voltage; however this system is not yet available in routine clinical practice [49].

Finally, the use of second- or third- generation DSCT scanners with high temporal resolution (75 ms or 66 ms, respectively) could help discriminate between motion artifacts due to irregular or high heart rates and true perfusion defects, avoiding false positive findings [19].

### 3.3. Dynamic CTP Imaging

The only CT based technology that permits absolute quantification of myocardial perfusion is dynamic CTP imaging. It is based on repeated acquisition of the myocardial tissue during the first-pass contrast uptake to create time-attenuation curves (TACs) for the region of interest (ROI) [53], providing more objective and reproducible assessment of myocardial iodine distribution in a similar way of positron emission tomographic (PET) perfusion imaging [37].

Hemodynamic parameters, such as the myocardial blood flow (MBF), MBF ratio, and myocardial blood volume (MBV), and semiquantitative parameters such as the up-slope, peak enhancement, time to peak (TTP), tissue transit time (TTT), and area under the curve (AUC) are derived by dedicated algorithms of these TACs (most of which are based on deconvolution methods already used in CMR studies) [54, 55].

**Figure 3:**
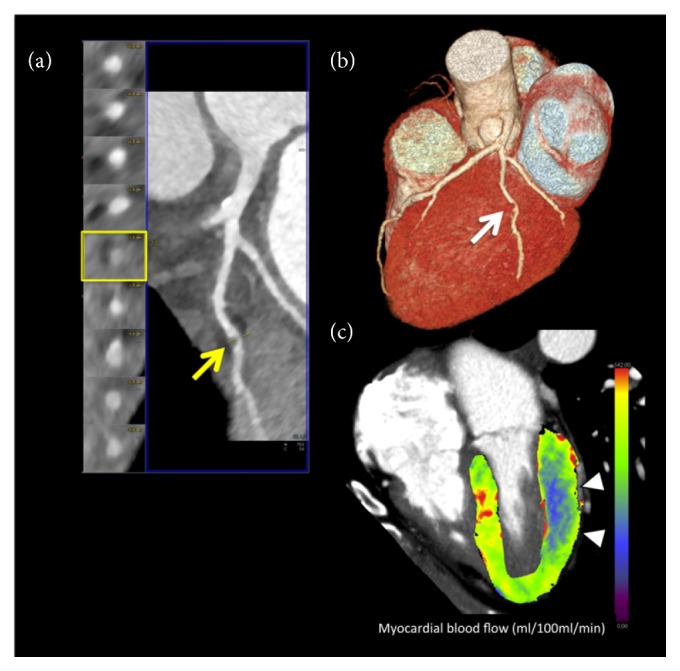
**Dynamic CTP imaging**. 67-year-old obese female patient with history of hyperlipidemia and smoking with suspected coronary artery disease. (a) Curved multiplanar reformation of coronary CT angiography data showed eccentric noncalcified plaque of the main obtuse marginal branch (OM) causing focal critical stenosis (>70% luminal narrowing), arrow. (b) Three-dimensional volume-rendering reconstruction confirmed the severe coronary artery stenosis of the OM (arrow). (c) Three-dimensional color-coded 4-chamber CT perfusion map image derived from the time-resolved dynamic acquisition during stress with the shuttle mode shows extensive perfusion defects in the territory of the OM (basal-middle lateral wall), color-coded in blue, arrowheads. The colors of the myocardium are coded according to the flow values with red, green, and yellow representing higher flow values than blue. The corresponding value of the hemodynamic parameters derived from the time-attenuation curves (TACs) demonstrates a significant reduction of myocardial blood flow in the territory of the OM, consistent with inducible ischemia. Absolute myocardial blood flow was 61.6 mL/100 mL/min and 118.2 mL/100 mL/min in the OM and remote myocardium (septal wall) territories, respectively.

Recently, the introduction of semiautomated three-dimensional software allowed a substantial reduction of the postprocessing phase, making the dynamic CTP more suited to routine clinical practice [34], Figure 3.

Whole-heart spatial coverage with appropriate temporal resolution is crucial to obtain multiple consecutive images at high heart rates [37].

Dynamic datasets acquisition is currently performed with two different approaches. The first one provides the use of single-tube multidetector CT scanners with 256 or 320 detector rows, which cover the whole cardiac volume while the table is stationary (detector Z-coverage is 78 or 160 mm, respectively). An alternative approach is second- and third-generation DSCT scanners, able to perform dynamic CTP imaging: by moving the scanner table back and forth (“shuttle mode”) between two scanning positions; it is possible to achieve a coverage of 73 or 105 mm, respectively, for the second- and third-generation DSCT scanners [34].

In both cases, image acquisition is performed during the systolic phase of the cardiac cycle when apical-basal length is shorter and myocardial wall is at maximal thickness.

Systolic phase length is relatively constant (about 200 ms) even when heart rate is high and irregular, so, images acquired in systole are less vitiated to beam-hardening artifacts because the amount of contrast medium needed for this phase is lower (typically 50 mL of contrast medium followed by 50 mL of saline at 5-6 mL/s is required) [53].

The obstacles to the clinical routine application of dynamic CTP are high radiation exposure, the relatively long breath-hold (approximately 30 sec) necessary for whole cardiac volume scanning, and spatial misalignment from two separated table positions when shuttle mode is used [19].

The use of motion correction and beam-hardening correction algorithms could minimize artifacts, improving image quality and diagnostic accuracy [34].

Advantages and disadvantages of static and dynamic CTP imaging are summarized in Table 1.

## 4. Accuracy of CTP Imaging

Many clinical studies and the first prospective multicenter trials have established the clinical feasibility and the diagnostic accuracy of static and dynamic CTP compared to SPECT, stress MRI, and/or invasive coronary angiography with and without fractional flow reserve (FFR), Tables 2–4.

A recent meta-analysis including 1188 patients in 19 studies showed that static CTP imaging in case of suspicion of known CAD, had a good agreement with SPECT and stress MRI perfusion with a sensitivity and specificity of 85% and 81%, respectively. When ICA was used as reference standard alone or in combination with SPECT or FFR, combined coronary CTA and CTP compared to coronary CTA alone significantly increased the specificity from 62% to 84% without significant decrease in sensitivity [79].

Similar results were obtained in a meta-analysis of Takx et al. [80] evaluating the diagnostic accuracy of different stress myocardial perfusion imaging modalities for the diagnosis of hemodynamically significant CAD compared to ICA with FFR as a reference standard. Takx et al. showed that the performance of CTP imaging was comparable to that of PET and stress MRI and substantially higher than that of SPECT and echocardiography, with a pooled sensitivity of 88% and specificity of 80% [80]. This finding was noted at both the vessel and the patient level. Furthermore, CTP showed a higher sensitivity than SPECT (88% versus 74%, respectively), because of a small number of false negative results [80].

A prospective multicenter international trial, the CORE 320 study (n=381), has confirmed that static CTP imaging has a higher accuracy in comparison with SPECT in terms of significant CAD (≥ 50%) detection, using as reference ICA [61]. The better performance of CTP imaging was due in part to its higher sensitivity in the detection of left main and multivessel CAD and in part to its superior spatial resolution, which permits a better evaluation of small subendocardial defects [61].

The increased sensitivity of CTP also derives from the more favorable extraction characteristics of iodinated contrast material allowing for a linear relationship between CT-derived metrics and myocardial blood flow. Conversely, Technetium-based tracers show a nonlinear net-tracer uptake in particular in the higher coronary flow range, causing the well-known-roll-off phenomenon [61].

The CORE 320 studies have also proved that the specificity and overall accuracy of coronary CTA in detecting significant CAD (≥50%) defined by ICA and SPECT are significantly increased by the addition of CTP at both the patient and vessel levels [60, 63]. This finding has been shown in patients with as well as without known CAD [60, 63].

Another recent randomized, multicenter, multivendor CTP study with regadenoson (n=110) by Cury et al. has shown that regadenoson-CTP imaging improved the diagnostic accuracy of coronary CTA from 69% to 85%, in particular by reducing the rate of false positive CTA results [64].

Moreover, CTP showed a high sensitivity and specificity of 90% and 84%, respectively, for the detection of myocardial ischemia as defined by a reversible perfusion defect in ≥ 2 myocardial segments on SPECT, with an agreement rate of 87% [64].

A considerable increase in diagnostic performance has also been largely proved when dual-energy myocardial CTP was coupled to coronary CTA, especially in terms of specificity, Table 3.

According to Meinel et al. the rest-stress protocol should be the first choice for evaluation of the myocardial blood supply in dual-energy CTP, with a sensitivity of 99%, specificity of 97%, positive predictive value (PPV) of 92%, and NPV of 100% using SPECT as reference of standard [66].

The DECIDE-Gold, an ongoing prospective multicenter study, will define the diagnostic accuracy of dual-energy to detect hemodynamic significant CAD, comparing it to fractional flow reserve (FFR) as a reference standard [81].

The available published data seem to suggest that static dual-energy and quantitative dynamic CTP imaging have a higher sensitivity, with respect to standard static monoenergetic CTP [34, 53], Tables 3 and 4. This might be due to the easiest detection of small perfusion defects from the quantitative analysis that cannot be appreciated by visual qualitative perfusion analysis of static CTP [53].

Stress dynamic myocardial CTP has been initially studied in preclinical trials demonstrating a good correlation of CT-derived perfusion values with microsphere derived MBF data, histopathology, and invasive measurements of coronary blood flow and FFR, Table 5.

It is important to note that, as reported by a recent large animal study, dynamic CTP has a superior discriminatory power in detecting myocardial ischemia than static first-pass CTP, using fluorescent microspheres as a reference standard for MBF [34, 84]. A significant difference in accuracy was noted at lower degree of stenosis (50%), demonstrating a higher sensitivity of dynamic CTP for the detection of subtle differences of myocardial perfusion as compared to single phase CTP acquisition [84].

Clinical researches have demonstrated that dynamic stress CTP may improve the PPV and specificity of coronary CTA alone [77, 78], especially for interpretation of the hemodynamic impact of intermediate-grade stenosis (30-70%) by using invasive FFR as the reference standard [78].

This modality also enables the quantification of the absolute value of coronary flow reserve (CFR) calculated as the ratio of hyperemic to baseline MBF with a high degree of correlation to SPECT [33, 54].

Moreover, dynamic CTP is useful in the global quantitative evaluation of left ventricular myocardial perfusion, especially in case of balanced ischemia caused by multivessel CAD [86].

According to quantitative PET and CTP studies, the relative MBF (an absolute MBF-to-remote MBF ratio) leads to better detection of hemodynamically significant coronary stenosis than does the absolute MBF derived from dynamic CTP imaging, probably reducing the impact of microvascular resistance on myocardial perfusion [87–89].

Semiquantitative parameters such as the TPR and myocardial reserve index (defined as the ratio of hyperemic and resting blood flow) have been suggested for static and dynamic myocardial CTP; however, they have a lower diagnostic accuracy than qualitative analysis by standard visual assessment [19, 57, 90].

The CATCH-2 (CArdiac cT in the treatment of acute CHest pain 2), a prospective randomized controlled multicenter study published in 2017, has showed the usefulness of myocardial CTP assessment in addition to CTA, in patients with recent acute-onset chest pain when acute coronary syndrome had been excluded, and who had a clinical indication for outpatient noninvasive testing [91]. Coupling CTA with CTP, the amount of patients with suspected CAD requiring invasive examination and treatment decreases [91].

Finally, as proved by a CATCH-trial substudy, myocardial CTP parameters predict mid-term clinical outcome in patients with recent acute-onset chest pain independently of the pretest probability of obstructive CAD [92]. Interestingly, patients with an ischemic burden involving >10% of the LV myocardium demonstrated the poorest prognosis [92].

## 5. Radiation Exposure

During the last years, CT scanners with higher spatial resolution (approximately 1/3 of millimeter), temporal resolution (up to 66 ms), and wider detector array (up to 320-detector row) were developed, with a substantial improvement in CT performance and reduction of radiation exposure [19]. Furthermore, the introduction of ECG-driven tube current modulation, BMI-adapted tube voltage modulation, and prospective ECG-triggered sequential scanning combined with advanced iterative image reconstruction algorithms has achieved 30-90% reductions in patient radiation exposure while guaranteeing the image quality [19].

Consequently, the contemporary estimated effective dose of coronary CTA and myocardial static CTP imaging will typically range between approximately 1.5 and 5.0 mSv, with an effective dose even to sub-millisievert levels for some exams [19, 93].

However, numerous factors may influence the radiation dose, such as patient's characteristics (BMI, cardiac output, and heart rate), the type of CT equipment available, and the CT protocols used, which has to be tailored to the patient. Despite these promising innovations, the relatively high radiation exposure during dynamic CTP acquisitions remains a problem to be solved since it acquires a series of multiple low-dose acquisitions for the generation of TACs. Recent data have demonstrated that the average radiation exposure of dynamic CTP imaging is greatly depending on protocol optimization with an average value of 9.23 mSv (versus 5.93 mSv for static CTP) [34], which is favorably comparable with that of traditional nuclear imaging approaches [53].

However, Hubbard et al. validated a low-dose dynamic CTP technique based on a first-pass analysis model by using only 2 volume scans as compared with standard protocol based on multiple acquisitions in an animal model, showing good correlation with invasive FFR at different stenosis severity reaching overall effective radiation doses of 2.64 mSv [94].

So current efforts are directed towards further reducing radiation exposure while maintaining a high diagnostic performance. In this regard, the use of recent technical innovations, including the low voltages (70 kV to 80 kV) acquisition, automated tube current modulation, and iterative reconstruction, seems to be able to achieve this ambitious goal [19].

## 6. Comparison with Other Noninvasive Techniques for Myocardial Perfusion Imaging

Many noninvasive techniques can perform an evaluation of myocardial perfusion, including SPECT, stress MRI, stress echocardiography, and positron emission tomography (PET) [95]. Nuclear imaging techniques such as PET and SPECT are established modalities for myocardial perfusion evaluation. These techniques are able to evaluate also myocardial viability and function but provide limited information regarding anatomy [96].

PET is the gold standard for absolute quantification of myocardial perfusion particularly when 13N-ammonia is used and may be superior to SPECT in spatial resolution, image quality, and diagnostic accuracy [96]. However, SPECT is more widely available and cheaper than PET, and the radionuclides are easier to prepare and less expensive and have longer half-lives compared to PET; thus this approach is more suitable in daily clinical routine [97]. SPECT is an excellent noninvasive modality for the diagnosis of CAD with a sensitivity of 87-89% and specificity of 73-75%, depending on the radionuclide and stress protocol [19, 37]. Additionally, SPECT may provide a refinement of risk stratification and has an independent prognostic value in different clinical settings such as stable CAD, prior to noncardiac surgery, after coronary revascularization, and in acute coronary syndromes [93]. Furthermore, recent advances in SPECT technology, including cadmium-zinc-telluride (CZT) semiconductor detector material, may allow absolute MBF measurements by SPECT but have yet to be implemented in clinical practice [98].

These observations have fueled the pursuit of hybrid imaging strategies in which radionuclide myocardial perfusion imaging is combined with coronary CTA. While promising, this approach has some important disadvantages including higher radiation doses and elevated costs [99].

Moreover, important SPECT limitations are the underestimation of the true extent of disease in patients with multivessel CAD and the photon attenuation artifacts typically due to breasts in women and diaphragm in men [99].

MRI is the most versatile imaging modality: it can be used for morphology, function, viability, and quantitative myocardial perfusion assessment [100]. Stress perfusion MRI performs better than SPECT for diagnosis of obstructive CAD, as reported in two large prospective randomized studies (MR-IMPACT and CE-MARC trials), and yields a similar diagnostic accuracy as PET, with a poll sensitivity of 89% and specificity of 76% [100]. Moreover spatial resolution of perfusion MRI (1-2 mm) is superior to that of SPECT, especially for the detection of subendocardial perfusion abnormalities [37]. Despite these excellent features, limitations to the clinical routine implementation of MRI perfusion assessment are the time-consuming image acquisition, the limited accessibility, and lack of widespread competence in cardiac MRI [37, 100].

Stress echocardiography is a well-established, real-time imaging modality with advantages including lack of radiation exposure, versatility, and affordability. Dobutamine stress echocardiography could provide information about ischemic abnormal ventricular wall motion but this modality is lower than dobutamine stress MRI in terms of specificity (87.5% versus 72.9%), negative predictive value (80.8% versus 67.3%), and overall diagnostic accuracy (80.4% versus 72%) [97].

The introduction of ultrasound contrast agents (microbubbles) has optimized the detection of RWMA and has enabled simultaneous assessment of left ventricular perfusion and viability, improving the sensitivity of the technique [100].

According to a large multicenter prospective trial, myocardial contrast echocardiography (MCE) has higher sensitivity but lower specificity compared to SPECT for CAD evaluation [101]. The superior sensitivity of MCE was independent of the severity of CAD and was especially evident in case of single vessel disease [101]. The major disadvantages of echocardiography are the well-known operator and reader dependence and the intrinsic technical limitations related to artifacts and poor thoracic imaging window, resulting in uninterpretable images in 10% of cases [100, 102].

Although functional information provided by any of these techniques is well-validated and extremely useful, none of them provide a comprehensive anatomical-functional evaluation within the same study. Currently, myocardial CTP imaging is the only noninvasive modality that allows quantifying coronary stenosis and determining its functional relevance, rendering it a potential “one-stop-shop” method for the diagnosis and global management of patients with ischemic heart disease [53].

Moreover, the imaging matrix of 512 × 512 pixels with an isotropic high spatial resolution (approximately 0.3 mm) of CTP is superior to nuclear imaging and enables evaluation of transmural differences in myocardial blood flow [100].

Moreover, CTP imaging using the iodinated contrast agent does not suffer of the nonlinear relationship between myocardial signal intensity and gadolinium contrast concentration, which might affect the accuracy of quantitative analysis of MBF in MRI perfusion imaging [103].

Finally, the wide availability of modern CT scanners makes CTP more accessible compared with other noninvasive tools, such as MRI or PET imaging [19].

Advantages and disadvantages of functional imaging with echo, SPECT, MRI, and CTP imaging are reported in Table 6.

## 7. CTP Imaging versus Noninvasive FFR (FFR_CT_)

Recently, a new technique to allow for noninvasive calculation of FFR based on conventional coronary CTA data (FFR_CT_) using computational fluid dynamics has been clinically validated [104].

In a recent study by Yang et al. [105], the combinations of static CTP imaging with coronary CTA and FFR_CT_ with CTA improved diagnostic performance compared with CTA alone. However, in the highest tertile of calcium score, specificity and positive predictive value of FFR_CT_ were significantly lower than those of first-pass CTP.

Accordingly, a combined approach of dynamic CTP imaging and FFR_CT_ has been demonstrated to improve diagnostic performance in detecting functional relevance CAD in comparison with invasive FFR [106]. For various reasons, it is unlikely that in clinical practice both CTP and FFR_CT_ techniques will be routinely applied in each patient. The best strategy in the future could be a stepwise approach, reserving CTP for intermediate FFR_CT_ results. This approach has been demonstrated to improve diagnostic performance while omitting nearly one-half of the population from dynamic CTP examinations [106].

The PERFECTION study (comparison between stress cardiac computed tomography PERfusion versus Fractional flow rEserve measured by Computed Tomography angiography In the evaluation of suspected cOroNary artery disease) will compare the diagnostic performance of an FFRCT-guided strategy to stress CTP for the detection of functionally significant CAD, using invasive FFR as the reference standard [107].

## 8. Discussion

### 8.1. Strengths, Limits, and Future Perspectives

The current evidence suggests that myocardial CTP imaging improves diagnostic accuracy of coronary CTA alone mainly by reducing the number of false positive findings, even when compared with invasive FFR.

With respect to this issue, an integration of both anatomical and physiological assessment of CAD may be a more robust “gatekeeper” to ICA by increasing the diagnostic accuracy while maintaining higher sensitivity compared to anatomical assessment alone. This may be particularly useful in difficult-to-interpret situations, such as in patients with coronary stents and heavily calcified coronary arteries in which blooming artifacts can hamper lumen visualization and correct stenosis measurements. Accordingly, recent studies have shown that stress CTP improves diagnostic performance in patients with a high Agatston calcium score [108] or coronary artery stents [109].

The utility of hemodynamic assessment by the integration of CTP and coronary CTA may have a potential role in stratifying cardiovascular risk and in the decision-making for the optimal medical intervention, although this potential role warrants further investigation.

Furthermore, in line with PET imaging, dynamic CTP imaging offers the ability to obtain quantitative data of hemodynamic parameters (such as MBF and MBV) and the assessment of absolute CFR. The combination of coronary CTA and dynamic myocardial CTP makes CT a very promising technique to evaluate patients with microvascular dysfunction because it not only reveals the absence of demonstrable obstructive CAD but also provides data about CFR, the current gold standard for clinically assessing microvascular function.

Quantification of hemodynamic parameters may be particularly useful for evaluation of specific patient population, such as patients with multivessel CAD, extensive nonobstructive CAD, hypertension, and diabetes mellitus [110].

When global myocardial ischemia exists due to multivessel CAD, it may be difficult to achieve an accurate diagnosis with the qualitative analysis method by static CTP. Conversely, MBF analysis may be able to identify multivessel disease and predict the extent of ischemia more accurately than static CTP imaging [34, 53].

The ability to quantify absolute MBF with dynamic stress CTP imaging permits identification of patients in whom the relative regional distribution of contrast agent may appear normal because of a balanced reduction of blood flow. Moreover, in patients with diffuse nonobstructive epicardial disease but no significant stenosis, the combination of plaque analysis by coronary CTA and CFR assessment derived by stress/rest dynamic CTP imaging may be helpful in identifying hemodynamic relevant coronary plaques, although not yet obstructive, and to avoid ascribing patient's symptoms to microvascular disease [111]. In fact, besides luminal area stenosis, other coronary plaque morphology and composition parameters may affect downstream myocardial perfusion. Accordingly, lesion-specific morphological features such as positive remodeling and noncalcified plaque volume have been associated with detrimental downstream hyperemic myocardial perfusion and FFR, independent of lesion severity, and are strong predictors of major cardiovascular events [112–114].

Furthermore, MBF analysis might also be advantageous in monitoring disease progression or perfusion changes in response to therapy such as for PET and MRI imaging [103, 115], although this potential application has still to be evaluated.

However, important considerations have to be highlighted when interpreting quantitative measurements of dynamic CTP. A substantial underestimation of absolute MBF from dynamic CTP has been reported, with a significant influence of CT-derived MBF by temporal sampling rate [33, 54, 94, 116, 117]. This may be related to the assumption of most modeling of dynamic CTP techniques that blood volume during the passage remains relatively constant. However, using iodinated contrast material blood volume actually increases [33]. In addition some contrast material may actually leave the intravascular space and enter the interstitium during the measurement time [33].

Finally, it is well-known that all iodinated contrast agents have an immediate and direct vasodilatory effect [36]. All these factors may explain the underestimation of maximal MBF by CTP imaging, although rest and hyperemic flow in the CTP studies are within the documented range of that in PET studies [34].

Moreover, the reported optimal MBF cutoff values for the differentiation of normal and ischemic myocardium varied considerably between dynamic CTP studies, ranging from 75 mL/100 mL/min to 103.1 mL/100 mL/min with a dual-source CT scanner and as high as 164 mL/100 mL/min using a 256-slices CT scanner [19, 34]. This broad range of cutoff values may be related to study design, pathophysiological and methodological factors, technical issues (different scanner technology, scanning protocols, and mathematical algorithms), patient risk profile, prevalence of CAD, sample sizes, and the used reference standard.

Moreover, numerous individual factors such as age, gender, race, BMI, presence and severity of CAD, the status of the microvasculature, individual adaptive vasodilator responsiveness, and/or the presence of collateral flow may affect MBF [19, 78, 87, 118].

Accordingly, considerable regional heterogeneity of the myocardial perfusion across coronary territories has been demonstrated in healthy and low-risk subjects [54, 119].

Large inter- and intraindividual differences in MBF distribution are already known from PET and MRI studies [19, 34]. Therefore large databases on normal perfusion values such as for nuclear imaging are needed to assure accurate clinical interpretation of quantitative perfusion values [53].

However, a major limit of dynamic CTP is the higher dose profile respect to static CTP due to the time-resolved acquisition of multiple phases.

Furthermore, CTP imaging may be affected by several artifacts, such as partial volume, beam hardening, breathing, and motion artifacts. In particular, the patient's breathing motion poses a major challenge for dynamic CTP, which requires a long acquisition time of approximately 30 s. Furthermore, the sequence “shuttle mode” implemented with the second-generation dual-source CT scanner to dynamically cover the entire left ventricle myocardium may be a source of motion artifact influencing the estimation of MBF derived from the TACs. Beam-hardening artifacts arise from the polychromatic nature of the X-rays in the CT acquisitions and the presence of high-density iodine contrast agent in the heart chambers, which results in a hypoattenuated shadowing artifact [47]. Areas affected by beam hardening can be misinterpreted as perfusion defects with a false positive finding artifact [47]. A potential strategy to overcome this limitation is to acquire dynamic images during the end-systolic phase when the volume of LV contrast agent is less [19].

Furthermore, some of these artifacts may be partially attenuated by well-validated beam hardening and motion correction algorithms implemented with latest CT scanner technology [19, 34].

Moreover, in most of the CTP studies, anti-ischemic drugs such as beta-blockers have not been withheld prior to stress testing; this may negatively affect the accuracy of CTP by decreasing the severity and the extent of myocardial perfusion defects. However, it is expected that the diagnostic performance of CTP imaging performed in patients without background medications could be even higher than reported.

Finally, other limits are the broad spectrum of clinical characteristics of the studied populations and the difficult to standardize the CTP imaging due to the heterogeneity of scanner manufacturers, acquisition protocols, stress protocols, image analysis algorithms, and postprocessing parameters.

In addition, no large-scale multicenter studies have demonstrated the clinical value of CTP imaging. Further researches with larger sample size and improved standardization of CTP imaging technique are warranted.

## 9. Conclusions 

Current evidence suggests that adding CTP imaging is a safe and powerful tool to improve the accuracy and the positive predictive value of coronary CTA alone because it not only provides anatomic information concerning luminal stenosis, plaque morphology, and total plaque burden but also provides data on myocardial tissue hemodynamics.

Different acquisition protocols for CTP imaging are available, which can assess myocardial perfusion in a qualitative, semiquantitative, or quantitative manner, with their own advantages and disadvantages.

In conclusion, coronary CTA combined with myocardial CTP imaging hold immense potential to evaluate almost every aspect of the broad spectra of ischemic heart disease with the possibility of guiding treatment decisions for a patient on an individual basis. Further researches with larger sample size should be designed and implemented to decide whether to adopt this new diagnostic modality in a routine clinical setting.

Finally, prognostic studies are needed to assess if this combined approach will likely have substantial impact on treatment costs, patient management, and outcome. The time to challenge this hypothesis with randomized prospective trials has come.

## Figures and Tables

**Table 1:** Main differences between static and dynamic CTP imaging.

	**STATIC CTP**	**DYNAMIC CTP**
**BREATH HOLD**	Shorter	Longer (about 30 sec)

**WALL MOTION EVALUATION**	Yes	No

**MYOCARDIAL PERFUSION QUANTIFICATION**	No	Yes

**RADIATION EXPOSURE**	+/++	++/+++

**Table 2:** Static single-energy CTP imaging.

**Author** **(Year)**	**Patients No.**	**CT Scanner**	**CTP Protocol**	**Stress Agent** **(dose)**	**Analysis**	**Reference Standard **	**SE (%)**	**SP (%)**	**PPV ** **(%)**	**NPV ** **(%)**	**Average CT Dose**
Blankstein et al [42] (2009)	34	DS (1st)	$\underset{\left(Retrosp\right)}{STRESS}\;/\;\underset{\left(Prosp\right)}{REST}\;/\underset{\left(Prosp\right)}{LE}$	Adenosine (0.14 mg/kg/min)	Visual	SPECT (for vessel)	84	80	71	90	9.1 mSv

Rocha-Filho et al [43] (2010)	35	DS (1st)	$\underset{\left(Retrosp\right)}{STRESS}\;/\;\underset{\left(Prosp\right)}{REST}$	Adenosine (0.14 mg/kg/min)	Visual	QCA (for vessel)	91	91	86	93	9.8 mSv

Feuchtner et al [46] (2011)	30	DS (2nd)	$\underset{\left(Prosp\right)}{STRESS}\;/\;\underset{\left(Prosp\right)}{REST}$	Adenosine (0.14 mg/kg/min)	Visual	MRI 1.5 T (for vessel)	96	88	93	94	0.93 mSv

Cury et al [56] (2011)	26	64-slice	$\underset{\left(Retrosp\right)}{STRESS}\;/\;\underset{\left(Retrosp\right)}{REST}$	Dypiridamole (0.56 mg/Kg)	Visual	SPECT (for patient)	94	78	89	87	14.4*∗* mSv

Ko et al [57] (2012)	40	320-slice	$\underset{\left(Prosp\right)}{REST}\;/\;\underset{\left(Prosp\right)}{STRESS}$	Adenosine (0.14 mg/kg/min)	TPR Visual^§^	FFR (for vessel) FFR (for vessel)	74 87	66 95	56 89	81 94	4.5 mSv

George et al [44] (2012)	50	320-slice	$\underset{\left(Prosp\right)}{REST}\;/\;\underset{\left(Prosp\right)}{STRESS}\;/\underset{\left(Prosp\right)}{LE}$	Adenosine (0.14 mg/kg/min)	TPR	SPECT (for patient)	72	91	81	85	7.0 mSv

Nasis et al [45] (2013)	20	320-slice	$\underset{\left(Prosp\right)}{REST}\;/\;\underset{\left(Prosp\right)}{STRESS}$	Adenosine (0.14 mg/kg/min)	Visual^§^	QCA + SPECT (for patient)	94	98	94	98	4.8 mSv

Bettencourt et al [58] (2013)	101	64-slice	$\underset{\left(Retrosp\right)}{STRESS}\;/\;\underset{\left(Prosp\right)}{REST}$	Adenosine (0.14 mg/kg/min)	Visual^§^	FFR (for patient) MRI 1.5 T (for patient)	89 67	83 95	80 91	90 78	5.0*∗* mSv

Wong et al [59] (2014)	75	320-slice	$\underset{\left(Prosp\right)}{REST}\;/\;\underset{\left(Prosp\right)}{STRESS}$	Adenosine (0.14 mg/kg/min)	Visual + TAG^§^	FFR (for vessel)	97	84	76	98	4.8 mSv

Rochitte et al (CORE320 study) [60] (2014)	381	320-slice	$\underset{\left(Prosp\right)}{REST}\;/\;\underset{\left(Prosp\right)}{STRESS}$	Adenosine (0.14 mg/kg/min)	Semiq	QCA + SPECT (for patient)	80	74	65	86	5.31 mSv

George et al (CORE320 study) [61] (2015)	381	320-slice	$\underset{\left(Prosp\right)}{REST}\;/\;\underset{\left(Prosp\right)}{STRESS}$	Adenosine (0.14 mg/kg/min)	Semiq	QCA (for patient)	88	55	75	75	NA

Yang et al [62] (2015)	75	DS (2nd)	$\underset{\left(Retrosp\right)}{STRESS}\;/\;\underset{\left(Retrosp\right)}{REST}$	Adenosine (0.14 mg/kg/min)	Visual	FFR (for patient) FFR (for vessel)	89 80	86 95	96 92	63 87	6.5 mSv

Magalhaes et al (CORE320 study) [63] (2015)	381	320-slice	$\underset{\left(Prosp\right)}{REST}\;/\;\underset{\left(Prosp\right)}{STRESS}$	Adenosine (0.14 mg/kg/min)	Visual^§^	QCA + SPECT (for patient)	78	73	64	85	NA

Cury et al (Regadenoson crossover study) [64] (2015)	110	Multivendor	$\underset{\left(Retrosp\;or\;Prosp\right)}{STRESS}\;/\;\underset{\left(Retrosp\right)}{REST}$	Regadenoson (0.4mg)	Semiq	SPECT	90	82	53	97	17.7*∗* mSv

CTP, computed tomography perfusion; 1st and 2nd, first and second generation; CT, computed tomography; DS, Dual-Source scanner; FFR, fractional flow reserve; MRI, magnetic resonance imaging; PROSP, prospective ECG-gating acquisition; RETROSP, retrospective ECG-gating acquisition; NA, nonassessable; NPV, negative predictive value; PPV, positive predictive value; QCA, quantitative coronary angiography; SE, sensitivity; SP, specificity; Semiq, semiquantitative analysis using a stress score; SPECT, myocardial perfusion scan; TPR, transmural perfusion ratio; TAG, transluminal attenuation gradient; *∗*, global radiation dose of the stress-rest protocol; §, accuracy of CT perfusion integrated with the coronary anatomic data.

**Table 3:** Static dual-energy CTP imaging.

**Author** **(Year)**	**Patients No.**	**CT** **Scanner**	**CTP Protocol**	**Stress Agent** **(dose)**	**Analysis**	**Reference** **Standard**	**SE (%)**	**SP (%)**	**PPV (%)**	**NPV (%)**	**Dose**
Ko et al [65] (2012)	45	DS (1st)	$\underset{\left(Retrosp\right)}{CCTA\;SECT}/\;\underset{\left(Retrosp\right)}{STRESS\;DECT}\;/\;\underset{\left(Retrosp\right)}{REST\;DECT}$	Adenosine (0.14 mg/kg/min)	Iodine Map (for vessel)	QCA	89	74	80	85	5.7 mSv

Meinel et al [66] (2014)	55	DS (2nd)	$\underset{\left(Retrosp\right)}{STRESS\;DECT}\;/\;\underset{\left(Retrosp\right)}{REST\;DECT}\;/\;\underset{\left(Retrosp\right)}{LE\;DECT}$	Adenosine (0.14 mg/kg/min) Regadenoson (0.4 mg)	Iodine Map (for segment)	SPECT	99	97	92	100	7.1 mSv

Delgado et al [67] (2013)	56	DS (2nd)	$\underset{\left(Retrosp\right)}{STRESS\;DECT}$	Adenosine (0.14 mg/kg/min)	Iodine Map (for segment)	MRI 1.5T	76	99	89	98	5.2 mSv

Kido et al [68] (2014)	21	DS (1st-2nd)	$\underset{\left(NA\right)}{CCTA\;SECT}\;/\;\underset{\left(Retrosp\right)}{STRESS\;DECT}$	Adenosine (0.16 mg/kg/min)	Iodine Map (for vessel)^§^	QCA	67	92	84	82	7.7 mSv

Kim et al [69] (2014)	50	DS (2nd)	$\underset{\left(Retrosp\right)}{STRESS\;DECT}\;/\;\underset{\left(Retrosp\right)}{CCTA\;SECT}$	Adenosine (0.14 mg/kg/min)	Iodine Map (for segment)	MRI 1.5T	77	94	53	98	6.5 mSv

Ko et al [70] (2014)	40	DS (1st)	$\underset{\left(Retrosp\right)}{CCTA\;SECT}/\;\underset{\left(Retrosp\right)}{STRESS\;DECT}\;/\;\underset{\left(Retrosp\right)}{REST\;DECT}$	Adenosine (0.14 mg/kg/min)	Iodine Map (for vessel)	QCA + MRI 3T^§^	87	79	71	91	4.6 mSv

Ko et al [71] (2014)	100	DS (1st)	$\underset{\left(Retrosp\right)}{STRESS\;DECT}\;/\;\underset{\left(Retrosp\right)}{CCTA\;SECT}$	Adenosine (0.14 mg/kg/min)	Iodine Map (for vessel)	QCA + MRI 1.5T and 3T^§^	88	79	73	91	4.2 mSv

CTP, computed tomography perfusion; 1st and 2nd, first and second generation; CT, computed tomography; DS, Dual-Source scanner; CCTA, coronary computed tomography angiography; DECT, dual-energy computed tomography; SECT, single-energy computed tomography; RETROSP, retrospective ECG-gating acquisition; NA, nonassessable; MRI, magnetic resonance imaging; No., patients' number; NPV, negative predictive value; PPV, positive predictive value; QCA, quantitative coronary angiography; SE, sensitivity; SP, specificity; SPECT, myocardial perfusion scan; ^§^, accuracy of CT perfusion integrated with the coronary anatomic data.

**Table 4:** Dynamic CTP imaging in human study.

**Author** **(Year)**	**Patients** **No.**	**CT** **Scanner**	**CTP Protocol**	**Stress Agent** **(dose)**	**Analysis**	**Reference** **Standard**	**SE (%)**	**SP (%)**	**PPV (%)**	**NPV (%)**	**Average CT** **Dose**
Bastarrika et al [72] (2010)	10	DS (2nd)	$\underset{\left(Prosp\right)}{CCTA}\;/\underset{\left(Shuttle\;mode\right)}{STRESS}$	Adenosine (0.14 mg/kg/min)	Visual Semiq Quantitative MBF	MRI (segment)	86	98	94	96	12.5 mSv

Ho et al [73] (2010)	35	DS (2nd)	$\underset{\left(Shuttle\;mode\right)}{STRESS}\;/\;\underset{\left(Prosp\right)}{CCTA}$	Dypiridamole (0.56 mg/Kg in 4′)	Quantitative MBF	SPECT (segment)	83	78	79	82	9.1 mSv

Bamberg et al [74] (2011)	33	DS (2nd)	$\underset{\left(Prosp\right)}{CCTA}\;/\underset{\left(Shuttle\;mode\right)}{STRESS}$	Adenosine (0.14 mg/kg/min)	Quantitative MBF	FFR^§^ (vessel)	93	87	75	97	10 mSv

Wang et al [75] (2012)	30	DS (2nd)	$\underset{\left(Prosp\right)}{CCTA}\;/\underset{\left(Shuttle\;mode\right)}{STRESS}$	Adenosine (0.14 mg/kg/min)	Visual Quantitative MBF and MBV	QCA + SPECT (vessel)	100	76	54	100	9.5 mSv

Greif et al [55] (2013)	65	DS (2nd)	$\underset{\left(Prosp\right)}{CCTA}\;/\underset{\left(Shuttle\;mode\right)}{STRESS}$	Adenosine (0.14 mg/kg/min)	Quantitative MBF	FFR (vessel)	95	74	49	98	9.7 mSv

Huber et al [76] (2013)	32	256-slice	$\underset{\left(Prosp\right)}{STRESS}$	Adenosine (0.14 mg/kg/min)	Quantitative MBF	FFR (vessel)	76	100	100	91	9.5 mSv

Bamberg et al [77] (2014)	31	DS (2nd)	$\underset{\left(Prosp\right)}{CCTA}\;/\underset{\left(Shuttle\;mode\right)}{STRESS}$	Adenosine (0.14 mg/kg/min)	Quantitative MBF and MBV	MRI 3T (vessel)	100	75	92	100	11.08 mSv

Rossi et al [78] (2014)	80	DS (2nd)	$\underset{\left(Prosp\right)}{CCTA}\;/\underset{\left(Shuttle\;mode\right)}{STRESS}$	Adenosine (0.14 mg/kg/min)	Quantitative MBF	FFR (vessel)	88	90	77	95	9.4 mSv

CTP, computed tomography perfusion; 2nd, second generation; CT, computed tomography; CCTA, coronary computed tomography angiography; PROSP, Prospective ECG-gating acquisition; DS, Dual-Source scanner; FFR, fractional flow reserve; MRI, magnetic resonance imaging; MBF, myocardial blood flow; MBV, myocardial blood volume; No., patients' number; NPV, negative predictive value; PPV, positive predictive value; QCA, quantitative coronary angiography; SE, sensitivity; Semiq, semiquantitative analysis; SP, specificity; SPECT, myocardial perfusion scan; ^§^, accuracy of CT perfusion integrated with the coronary anatomic data.

**Table 5:** Dynamic CTP imaging in animal studies.

**Author** **(Year)**	**No.**	**CT** **Scanner**	**CTP** **Protocol**	**Stress** **Agent** **(dose)**	**Reference** **Standard**	**Analysis**	**Dose **
Bamberg et al [82] (2012)	7 pigs	DS (2nd)	$\underset{\left(Shuttle\;mode\right)}{REST\;/\;STRESS}$	Adenosine (0.14 mg/kg/min)	Microsphere MBF	Quantitative MBF	10.6 (mSv)

Rossi et al [83] (2013)	7 pigs	DS (2nd)	$\underset{\left(Shuttle\;mode\right)}{STRESS}$	Adenosine (0.50 mg/kg/min)	CBF and FFR	Quantitative MBF	17.1 (mSv)

Schwarz et al [84] (2013)	6 pigs	DS (2nd)	$\underset{\left(Shuttle\;mode\right)}{REST\;/\;STRESS}$	Adenosine (0.14 mg/kg/min)	Microsphere MBF	Quantitative MBF + attenuation values (HU)	11.3 + 0.88 (mSv)

Bamberg et al [85] (2014)	12 pigs	DS (2nd)	$\underset{\left(Shuttle\;mode\right)}{REST\;/\;STRESS}$	Adenosine (0.14 mg/kg/min)	Histopathology Microsphere MBF	Quantitative MBF, MBV, K_trans_	NA

2nd, second generation; No., number of animals; CT, computed tomography; CBF, coronary blood flow; CTP, CT perfusion imaging; DS, Dual-Source scanner; FFR, fractional flow reserve; HU: Hounsfield Unit; Ktrans, permeability constant; MBF, myocardial blood flow; MBV, myocardial blood volume; NA, nonassessable.

**Table 6:** Major advantages and limitations of current noninvasive techniques for myocardial perfusion evaluation.

	**Advantages**	**Limitations**
**PET**	(i) Modality of choice for absolute myocardial perfusion quantification. (ii) Superior to SPECT in spatial and temporal resolution, image quality and diagnostic accuracy. (iii) Can be performed in patients with pacemakers or implantable cardioverter defibrillator.	(i) High cost. (ii) Radiation exposure. (iii) Not much available *⟶* more suitable in research setting then in clinical practice.

**SPECT**	(i) Radionuclides are easier to prepare, less expensive and have longer half-lives compared to PET *⟶* more suitable in daily clinical routine. (ii) High SE and high SP for detection of ischaemia. (iii) Allows evaluation of LV function. (iv) Very useful for risk stratification. (v) Provides important prognostic information in different clinical settings, especially in stable CAD.	(i) Radiation exposure. (ii) Relatively high cost and time consuming. (iii) Limited information regarding anatomy due to low spatial resolution. (iv) Photon attenuation artefacts (particularly in obese subjects) may produce FP. (v) In patients with multivessel disease, SPECT may underestimate the true extent of disease (balanced reduction in myocardial hyperaemic blood flow not detectable by semi-quantitative analysis) *⟶* prefer other modalities in patients with higher pre-test likelihood of multivessel CAD.

**MRI**	(i) Not require ionizing radiation. (ii) Higher SE and SP for detection of ischaemia than SPECT. (iii) High spatial resolution. (iv) Allows evaluation of LV function (v) Multiparametric imaging technique *⟶* strong role in differentiate ischaemic from non-ischaemic cardiac diseases. (vi) Provides important prognostic information.	(i) Time-consuming image acquisition (ii) Limited availability (iii) Lack of widespread expertise (iv) Common cardiac devices as pacemakers, implantable defibrillators, etc.. are still considered a contraindication to CMR. (v) Claustrophobia. (vi) Heart rate and respiratory motion artefacts.

**ECHO**	(i) Radiation-free. (ii) Rapid and safe *⟶*suitable technique as a first- line approach. (iii) Can be performed at the bedside. (iv) Less expensive than other modalities. (v) Provides simultaneous evaluation of perfusion and function in real time. (vi) Allows assessment of many non-ischemic cardiac diseases. (vii) MCE with microbubbles has superior spatial/temporal resolution and SE compared to SPECT.	(i) Poor thoracic window in at least 10% of patients. (ii) Operator and reader dependence. (iii) Artifacts.

**CTP**	(i) Provides integrated anatomic and functional evaluation in a single examination. (ii) Very fast exam. (iii) Widely available. (iv) High sensitivity and high specificity. (v) Superior submillimetre spatial resolution with respect to SPECT*⟶* detection of smaller, especially subendocardial, perfusion defects. (vi) Allows evaluation of important non-coronary cardiac findings. (vii) Provides important prognostic information.	(i) Radiation exposure, especially for dynamic CTPI (but still lower than nuclear imaging) (ii) Breath and beam hardening artifacts. (iii) High heart rate artifacts.

PET, positron emission tomography; SPECT, single photon-emission computed tomography; MRI, magnetic resonance imaging; ECHO, echocardiography; CTP, computed tomography perfusion imaging; SE, sensitivity; SP, specificity; CAD, coronary artery disease; LV, left ventricular; FP, false positive; MCE, myocardial contrast echocardiography.

## References

[1] McCollough C. H., Zink F. E. (1999). Performance evaluation of a multi-slice CT system. *Medical Physics*.

[2] Achenbach S., Ulzheimer S., Baum U. (2000). Noninvasive coronary angiography by retrospectively ECG-gated multislice spiral CT. *Circulation*.

[3] Yang L., Zhou T., Zhang R. (2014). Meta-analysis: diagnostic accuracy of coronary CT angiography with prospective ECG gating based on step-and-shoot, Flash and volume modes for detection of coronary artery disease. *European Radiology*.

[4] Mark D. B., Berman D. S., Budoff M. J. (2010). CCF/ACR/AHA/NASCI/SAIP/SCAI/SCCT 2010 expert consensus document on coronary computed tomographic angiography: a report of the American College of Cardiology Foundation Task Force on Expert Consensus Documents. *Circulation*.

[5] Fihn S. D., Blankenship J. C., Alexander K. P., Bittl J. A. (2014). 2014 ACC/AHA/AATS/PCNA/SCAI/STS focused update of the guideline for the diagnosis and management of patients with stable ischemic heart disease: a report of the American College of Cardiology/American Heart Association Task Force on Practice Guidelines, and the American Association for Thoracic Surgery, Preventive Cardiovascular Nurses Association, Society for Cardiovascular Angiography and Interventions, and Society of Thoracic Surgeons. *Circulation*.

[6] National Institute for Health and Care Excellence (2016). Chest pain of recent onset: assessment and diagnosis of recent onset chest pain or discomfort of suspected cardiac origin. *Clinical guideline 95*.

[7] Neglia D., Rovai D., Caselli C. (2015). EVINCI Study Investigators. Detection of significant coronary artery disease by noninvasive anatomical and functional imaging. *Circulation: Cardiovascular Imaging*.

[8] SCOT-HEART investigators (2015). CT coronary angiography in patients with suspected angina due to coronary heart disease (SCOT-HEART): an open-label, parallel-group, multicentre trial. *The Lancet*.

[9] Douglas P. S., Hoffmann U., Patel M. R., PROMISE Investigators (2015). Outcomes of anatomical versus functional testing for coronary artery disease. *The New England Journal of Medicine*.

[10] Maffei E., Seitun S., Palumbo A. (2011). Prognostic value of Morise clinical score, calcium score and computed tomography coronary angiography in patients with suspected or known coronary artery disease. *La Radiologia Medica*.

[11] Maffei E., Seitun S., Martini C. (2011). Prognostic value of computed tomography coronary angiography in patients with chest pain of suspected cardiac origin. *La radiologia medica*.

[12] Aldrovandi A., Maffei E., Seitun S. (2012). Major Adverse Cardiac Events and the Severity of Coronary Atherosclerosis Assessed by Computed Tomography Coronary Angiography in an Outpatient Population With Suspected or Known Coronary Artery Disease. *Journal of Thoracic Imaging*.

[13] Aldrovandi A., Maffei E., Palumbo A. (2009). Prognostic value of computed tomography coronary angiography in patients with suspected coronary artery disease: a 24-month follow-up study. *European Radiology*.

[14] Min J. K., Dunning A., Lin F. Y., CONFIRM Investigators (2011). Age- and sex-related differences in all-cause mortality risk based on coronary computed tomography angiography findings results from the International Multicenter CONFIRM (Coronary CT Angiography Evaluation for Clinical Outcomes: An International Multicenter Registry) of 23,854 patients without known coronary artery disease. *Journal of the American College of Cardiology*.

[15] Andreini D., Pontone G., Mushtaq S. (2012). A long-term prognostic value of coronary CT angiography in suspected coronary artery disease. *JACC: Cardiovascular Imaging*.

[16] Hoffmann U., Ferencik M., Udelson J. E. (2017). PROMISE Investigators. Prognostic Value of Noninvasive Cardiovascular Testing in Patients With Stable Chest Pain: Insights From the PROMISE Trial (Prospective Multicenter Imaging Study for Evaluation of Chest Pain). *Circulation*.

[17] van Werkhoven J. M., Schuijf J. D., Gaemperli O. (2009). Prognostic value of multislice computed tomography and gated single-photon emission computed tomography in patients with suspected coronary artery disease. *Journal of the American College of Cardiology*.

[18] Schuijf J. D., Wijns W., Jukema J. W. (2006). Relationship between noninvasive coronary angiography with multi-slice computed tomography and myocardial perfusion imaging. *Journal of the American College of Cardiology*.

[19] Seitun S., Castiglione Morelli M., Budaj I. (2016). Stress Computed Tomography Myocardial Perfusion Imaging: A New Topic in Cardiology. *Revista Española de Cardiología*.

[20] Iwasaki K. (2014). Myocardial ischemia is a key factor in the management of stable coronary artery disease. *World Journal of Cardiology*.

[21] Tonino P. A. L., Fearon W. F., de Bruyne B. (2010). Angiographic versus functional severity of coronary artery stenoses in the FAME study fractional flow reserve versus angiography in multivessel evaluation. *Journal of the American College of Cardiology*.

[22] de Bruyne B., Pijls N. H., Kalesan B. (2012). Fractional flow reserve-guided PCI versus medical therapy in stable coronary disease. *The New England Journal of Medicine*.

[23] Boden W., O’Rourke R., Teo K. (2007). Optimal medical therapy with or without PCI for stable coronary disease. *The New England Journal of Medicine*.

[24] Liga R., Vontobel J., Rovai D., EVINCI Study Investigators (2016). EVINCI Study Investigators. Multicentre multi-device hybrid imaging study of coronary artery disease: results from the EValuation of INtegrated Cardiac Imaging for the Detection and Characterization of Ischaemic Heart Disease (EVINCI) hybrid imaging population. *European Heart Journal Cardiovascular Imaging*.

[25] Maffei E., Martini C., Rossi A. (2012). Diagnostic accuracy of second-generation dual-source computed tomography coronary angiography with iterative reconstructions: A real-world experience. *La Radiologia Medica*.

[26] Maffei E., Martini C., Tedeschi C. (2012). Diagnostic accuracy of 64-slice computed tomography coronary angiography in a large population of patients without revascularisation: registry data on the comparison between male and female population. *La radiologia medica*.

[27] Maffei E., Martini C., De Crescenzo S. (2010). Low dose CT of the heart: a quantum leap into a new era of cardiovascular imaging. *La radiologia medica*.

[28] Kurata A., Mochizuki T., Koyama Y. (2005). Myocardial perfusion imaging using adenosine triphosphate stress multi-slice spiral computed tomography: Alternative to stress myocardial perfusion scintigraphy. *Circulation Journal*.

[29] Baxa J., Hromádka M., Šedivý J. (2015). Regadenoson-Stress Dynamic Myocardial Perfusion Improves Diagnostic Performance of CT Angiography in Assessment of Intermediate Coronary Artery Stenosis in Asymptomatic Patients. *BioMed Research International*.

[30] Xu L., Sun Z., Fan Z. (2015). Noninvasive physiologic assessment of coronary stenosis using cardiac CT. *BioMed Research International*.

[31] Pontone G., Andreini D., Baggiano A. (2015). Functional Relevance of Coronary Artery Disease by Cardiac Magnetic Resonance and Cardiac Computed Tomography: Myocardial Perfusion and Fractional Flow Reserve. *BioMed Research International*.

[32] Pontone G., Muscogiuri G., Andreini D. (2016). The New Frontier of Cardiac Computed Tomography Angiography: Fractional Flow Reserve and Stress Myocardial Perfusion. *Current Treatment Options in Cardiovascular Medicine*.

[33] Marini C., Seitun S., Zawaideh C. (2017). Comparison of coronary flow reserve estimated by dynamic radionuclide SPECT and multi-detector x-ray CT. *Journal of Nuclear Cardiology*.

[34] Cademartiri F., Seitun S., Clemente A. (2017). Myocardial blood flow quantification for evaluation of coronary artery disease by computed tomography. *Cardiovascular Diagnosis and Therapy*.

[35] Pelgrim G. J., Das M., Haberland U. (2015). Development of an Ex Vivo, Beating Heart Model for CT Myocardial Perfusion. *BioMed Research International*.

[36] Gould K. L., Lipscomb K., Hamilton G. W. (1974). Physiologic basis for assessing critical coronary stenosis. Instantaneous flow response and regional distribution during coronary hyperemia as measures of coronary flow reserve. *American Journal of Cardiology*.

[37] Varga-Szemes A., Meinel F. G., De Cecco C. N., Fuller S. R., Bayer R. R., Joseph Schoepf U. (2015). CT myocardial perfusion imaging. *American Journal of Roentgenology*.

[38] Johnson N. P., Gould K. L. (2015). Regadenoson versus dipyridamole hyperemia for cardiac PET imaging. *JACC: Cardiovascular Imaging*.

[39] Charoenpanichkit C., Hundley W. (2010). The 20 year evolution of dobutamine stress cardiovascular magnetic resonance. *Journal of Cardiovascular Magnetic Resonance*.

[40] Sicari R., Cortigiani L. (2017). The clinical use of stress echocardiography in ischemic heart disease. *Cardiovascular Ultrasound*.

[41] Keir M., Spears D., Caldarone C., Crean A. M. (2017). Proving the innocence of a “malignant” coronary artery: Calling dobutamine stress CT for the defence!. *Journal of Cardiovascular Computed Tomography*.

[42] Blankstein R., Shturman L. D., Rogers I. S. (2009). Adenosine-induced stress myocardial perfusion imaging using dual-source cardiac computed tomography. *Journal of the American College of Cardiology*.

[43] Rocha-Filho J. A., Blankstein R., Shturman L. D. (2010). Incremental value of adenosine-induced stress myocardial perfusion imaging with dual-source CT at cardiac CT angiography. *Radiology*.

[44] George R. T., Arbab-Zadeh A., Miller J. M. (2012). Computed tomography myocardial perfusion imaging with 320-row detector computed tomography accurately detects myocardial ischemia in patients with obstructive coronary artery disease. *Circulation: Cardiovascular Imaging*.

[45] Nasis A., Ko B. S., Leung M. C. (2013). Diagnostic accuracy of combined coronary angiography and adenosine stress myocardial perfusion imaging using 320-detector computed tomography: Pilot study. *European Radiology*.

[46] Feuchtner G., Goetti R., Plass A. (2011). Adenosine stress high-pitch 128-slice dual-source myocardial computed tomography perfusion for imaging of reversible myocardial ischemia comparison with magnetic resonance imaging. *Circulation: Cardiovascular Imaging*.

[47] Mehra V. C., Valdiviezo C., Arbab-Zadeh A. (2011). A stepwise approach to the visual interpretation of CT-based myocardial perfusion. *Journal of Cardiovascular Computed Tomography*.

[48] Kang S.-J., Yang D. H., Koo H. J. (2017). Intravascular ultrasound-derived morphological predictors of myocardial ischemia assessed by stress myocardial perfusion computed tomography. *Catheterization and Cardiovascular Interventions*.

[49] Danad I., Hartaigh B. Ó., Min J. K. (2015). Dual-energy computed tomography for detection of coronary artery disease. *Expert Review of Cardiovascular Therapy*.

[50] Yi Y., Jin Z. Y., Wang Y. N. (2016). Advances in myocardial CT perfusion imaging technology. *American Journal of Translational Research*.

[51] Danad I., Fayad Z. A., Willemink M. J., Min J. K. (2015). New applications of cardiac computed tomography: Dual-energy, spectral, and molecular CT imaging. *JACC: Cardiovascular Imaging*.

[52] Cannaò P. M., Joseph Schoepf U., Muscogiuri G. (2015). Technical prerequisites and imaging protocols for dynamic and dual energy myocardial perfusion imaging. *European Journal of Radiology*.

[53] Danad I., Szymonifka J., Schulman-Marcus J., Min J. K. (2016). Static and dynamic assessment of myocardial perfusion by computed tomography. *European Heart Journal—Cardiovascular Imaging*.

[54] Ho K.-T., Ong H.-Y., Tan G., Yong Q.-W. (2015). Dynamic CT myocardial perfusion measurements of resting and hyperaemic blood flow in low-risk subjects with 128-slice dual-source CT. *European Heart Journal of Cardiovascular Imaging*.

[55] Greif M., von Ziegler F., Bamberg F. (2013). CT stress perfusion imaging for detection of haemodynamically relevant coronary stenosis as defined by FFR. *Heart*.

[56] Cury R. C., Magalhães T. A., Paladino A. T. (2011). Dipyridamole stress and rest transmural myocardial perfusion ratio evaluation by 64 detector-row computed tomography. *Journal of Cardiovascular Computed Tomography*.

[57] Ko B. S., Cameron J. D., Leung M. (2012). Combined CT coronary angiography and stress myocardial perfusion imaging for hemodynamically significant stenoses in patients with suspected coronary artery disease: a comparison with fractional flow reserve. *JACC: Cardiovascular Imaging*.

[58] Bettencourt N., Ferreira N. D., Leite D. (2013). CAD detection in patients with intermediate-high pre-test probability: Low-dose ct delayed enhancement detects ischemic myocardial scar with moderate accuracy but does not improve performance of a stress-rest ct perfusion protocol. *JACC: Cardiovascular Imaging*.

[59] Wong D. T. L., Ko B. S., Cameron J. D. (2014). Comparison of diagnostic accuracy of combined assessment using adenosine stress computed tomography perfusion + computed tomography angiography with transluminal attenuation gradient + computed tomography angiography against invasive fractional flow reserve. *Journal of the American College of Cardiology*.

[60] Rochitte C. E., George R. T., Chen M. Y. (2015). Computed tomography angiography and perfusion to assess coronary artery stenosis causing perfusion defects by single photon emission computed tomography: the CORE320 study. *European Heart Journal*.

[61] George R. T., Mehra V. C., Chen M. Y. (2015). Myocardial CT Perfusion Imaging and SPECT for the Diagnosis of Coronary Artery Disease: A Head-to-Head Comparison from the CORE320 Multicenter Diagnostic Performance Study. *Radiology*.

[62] Yang D. H., Kim Y.-H., Roh J.-H. (2015). Stress myocardial perfusion CT in patients suspected of having coronary artery disease: Visual and quantitative analysis-validation by using fractional flow reserve. *Radiology*.

[63] Magalhães T. A., Kishi S., George R. T. (2015). Combined coronary angiography and myocardial perfusion by computed tomography in the identification of flow-limiting stenosis - The CORE320 study: An integrated analysis of CT coronary angiography and myocardial perfusion. *Journal of Cardiovascular Computed Tomography*.

[64] Cury R. C., Kitt T. M., Feaheny K. (2015). A randomized, multicenter, multivendor study of myocardial perfusion imaging with regadenoson CT perfusion vs single photon emission CT. *Journal of Cardiovascular Computed Tomography*.

[65] Ko S. M., Choi J. W., Hwang H. K., Song M. G., Shin J. K., Chee H. K. (2012). Diagnostic performance of combined noninvasive anatomic and functional assessment with dual-source CT and adenosine- induced stress dual-energy CT for detection of significant coronary stenosis. *American Journal of Roentgenology*.

[66] Meinel F. G., de Cecco C. N., Schoepf U. J. (2014). First-arterial-pass dual-energy CT for assessment of myocardial blood supply: do we need rest, stress, and delayed acquisition? Comparison with SPECT. *Radiology*.

[67] Delgado C., Vázquez M., Oca R., Vilar M., Trinidad C., Sanmartin M. (2013). Myocardial ischemia evaluation with dual-source computed tomography: comparison with magnetic resonance imaging. *Revista Española de Cardiología*.

[68] Kido T., Watanabe K., Saeki H. (2014). Adenosine triphosphate stress dual-source computed tomography to identify myocardial ischemia: comparison with invasive coronary angiography. *SpringerPlus*.

[69] Kim S. M., Chang S.-A., Shin W., Choe Y. H. (2014). Dual-energy CT perfusion during pharmacologic stress for the assessment of myocardial perfusion defects using a second-generation dual-source CT: A comparison with cardiac magnetic resonance imaging. *Journal of Computer Assisted Tomography*.

[70] Ko S. M., Park J. H., Hwang H. K., Song M. G. (2014). Direct comparison of stress- and rest-dual-energy computed tomography for detection of myocardial perfusion defect. *The International Journal of Cardiovascular Imaging*.

[71] Ko S. M., Song M. G., Chee H. K., Hwang H. K., Feuchtner G. M., Min J. K. (2014). Diagnostic performance of dual-energy CT stress myocardial perfusion imaging: direct comparison with cardiovascular MRI. *American Journal of Roentgenology*.

[72] Bastarrika G., Ramos-Duran L., Rosenblum M. A., Kang D. K., Rowe G. W., Schoepf U. J. (2010). Adenosine-stress dynamic myocardial CT perfusion imaging: initial clinical experience. *Investigative Radiology*.

[73] Ho K.-T., Chua K.-C., Klotz E., Panknin C. (2010). Stress and rest dynamic myocardial perfusion imaging by evaluation of complete time-attenuation curves with dual-source CT. *JACC: Cardiovascular Imaging*.

[74] Bamberg F., Becker A., Schwarz F. (2011). Detection of hemodynamically significant coronary artery stenosis: incremental diagnostic value of dynamic CT-based myocardial perfusion imaging. *Radiology*.

[75] Wang Y., Qin L., Shi X. (2012). Adenosine-stress dynamic myocardial perfusion imaging with second-generation dual-source CT: Comparison with conventional catheter coronary angiography and SPECT nuclear myocardial perfusion imaging. *American Journal of Roentgenology*.

[76] Huber A. M., Leber V., Gramer B. M. (2013). Myocardium: dynamic versus single-shot CT perfusion imaging. *Radiology*.

[77] Bamberg F., Marcus R. P., Becker A. (2014). Dynamic myocardial CT perfusion imaging for evaluation of myocardial ischemia as determined by MR imaging. *JACC: Cardiovascular Imaging*.

[78] Rossi A., Dharampal A., Wragg A. (2014). Diagnostic performance of hyperaemic myocardial blood flow index obtained by dynamic computed tomography: Does it predict functionally significant coronary lesions?. *European Heart Journal of Cardiovascular Imaging*.

[79] Sørgaard M. H., Kofoed K. F., Linde J. J. (2016). Diagnostic accuracy of static CT perfusion for the detection of myocardial ischemia. A systematic review and meta-analysis. *Journal of Cardiovascular Computed Tomography*.

[80] Takx R. A. P. (2015). Diagnostic accuracy of stress myocardial perfusion imaging compared to invasive coronary angiography with fractional flow reserve meta-analysis. *Circulation: Cardiovascular Imaging*.

[81] Truong Q. A., Knaapen P., Pontone G. (2015). Rationale and design of the dual-energy computed tomography for ischemia determination compared to “gold standard” non-invasive and invasive techniques (DECIDE-Gold): A multicenter international efficacy diagnostic study of rest-stress dual-energy computed tomography angiography with perfusion. *Journal of Nuclear Cardiology*.

[82] Bamberg F., Hinkel R., Schwarz F. (2012). Accuracy of dynamic computed tomography adenosine stress myocardial perfusion imaging in estimating myocardial blood flow at various degrees of coronary artery stenosis using a porcine animal model. *Investigative Radiology*.

[83] Rossi A., Uitterdijk A., Dijkshoorn M. (2013). Quantification of myocardial blood flow by adenosine-stress CT perfusion imaging in pigs during various degrees of stenosis correlates well with coronary artery blood flow and fractional flow reserve. *European Heart Journal of Cardiovascular Imaging*.

[84] Schwarz F., Hinkel R., Baloch E. (2013). Myocardial CT perfusion imaging in a large animal model: comparison of dynamic versus single-phase acquisitions. *JACC: Cardiovascular Imaging*.

[85] Bamberg F., Hinkel R., Marcus R. P. (2014). Feasibility of dynamic CT-based adenosine stress myocardial perfusion imaging to detect and differentiate ischemic and infarcted myocardium in an large experimental porcine animal model. *The International Journal of Cardiovascular Imaging*.

[86] Meinel F. G., Ebersberger U., Schoepf U. J. (2014). Global quantification of left ventricular myocardial perfusion at dynamic CT: Feasibility in a multicenter patient population. *American Journal of Roentgenology*.

[87] Kono A. K., Coenen A., Lubbers M. (2014). Relative myocardial blood flow by dynamic computed tomographic perfusion imaging predicts hemodynamic significance of coronary stenosis better than absolute blood flow. *Investigative Radiology*.

[88] Wichmann J. L., Meinel F. G., Schoepf U. J. (2015). Absolute versus relative myocardial blood flow by dynamic CT myocardial perfusion imaging in patients with anatomic coronary artery disease. *American Journal of Roentgenology*.

[89] Stuijfzand W. J., Uusitalo V., Kero T. (2014). Relative flow reserve derived from quantitative perfusion imaging may not outperform stress myocardial blood flow for identification of hemodynamically significant coronary artery disease. *Circulation: Cardiovascular Imaging*.

[90] Coenen A., Lubbers M. M., Kurata A. (2017). Diagnostic value of transmural perfusion ratio derived from dynamic CT-based myocardial perfusion imaging for the detection of haemodynamically relevant coronary artery stenosis. *European Radiology*.

[91] Sørgaard M. H., Linde J. J., Kühl J. T. (2017). Value of myocardial perfusion assessment with coronary computed tomography angiography in patients with recent acute-onset chest pain. *JACC: Cardiovascular Imaging*.

[92] Linde J. J., Sørgaard M., Kühl J. T. (2017). Prediction of clinical outcome by myocardial CT perfusion in patients with low-risk unstable angina pectoris. *The International Journal of Cardiovascular Imaging*.

[93] Gaemperli O., Saraste A., Knuuti J. (2012). Cardiac hybrid imaging. *European Heart Journal—Cardiovascular Imaging*.

[94] Hubbard L., Ziemer B., Lipinski J. (2016). Functional assessment of coronary artery disease using whole-heart dynamic computed tomographic perfusion. *Circulation: Cardiovascular Imaging*.

[95] Adams D., Hessel S., Judy P., Stein J., Abrams H. (1976). Computed tomography of the normal and infarcted myocardium. *American Journal of Roentgenology*.

[96] Qayyum A., Kastrup J. (2015). Measuring myocardial perfusion: the role of PET, MRI and CT. *Clinical Radiology*.

[97] Caruso D., Eid M., Schoepf U. J. (2016). Dynamic CT myocardial perfusion imaging. *European Journal of Radiology*.

[98] Nkoulou R., Fuchs T. A., Pazhenkottil A. P. (2016). Absolute myocardial blood flow and flow reserve assessed by gated SPECT with cadmium-zinc-telluride detectors using 99mTc-tetrofosmin: Head-to-head comparison with 13N-ammonia PET. *Journal of Nuclear Medicine*.

[99] Burrell S., MacDonald A. (2006). Artifacts and pitfalls in myocardial perfusion imaging. *Journal of Nuclear Medicine Technology*.

[100] Maffei E., Seitun S., Guaricci A. I., Cademartiri F. (2016). Chest pain: Coronary CT in the ER. *British Journal of Radiology*.

[101] Senior R., Becher H., Monaghan M., EACVI Scientific Documents Committee for 2014–16 and 2016–18, EACVI Scientific Documents Committee for 2014–16 and 2016–18 (2017). Clinical practice of contrast echocardiography: recommendation by the European Association of Cardiovascular Imaging (EACVI) 2017. *European Heart Journal Cardiovascular Imaging*.

[102] Dedic A., Genders T. S., Nieman K., Hunink M. G. M. (2013). Imaging strategies for acute chest pain in the emergency department. *American Journal of Roentgenology*.

[103] Lee D. C., Johnson N. P. (2009). Quantification of absolute myocardial blood flow by magnetic resonance perfusion imaging. *JACC: Cardiovascular Imaging*.

[104] Nørgaard B. L., Leipsic J., Gaur S. (2014). Diagnostic performance of noninvasive fractional flow reserve derived from coronary computed tomography angiography in suspected coronary artery disease: the NXT trial (Analysis of Coronary Blood Flow Using CT Angiography: Next Steps). *Journal of the American College of Cardiology*.

[105] Yang D. H., Kim Y., Roh J. H. (2017). Diagnostic performance of on-site CT-derived fractional flow reserve versus CT perfusion. *European Heart Journal Cardiovascular Imaging*.

[106] Coenen A., Rossi A., Lubbers M. M. (2017). Integrating CT Myocardial Perfusion and CT-FFR in the Work-Up of Coronary Artery Disease. *JACC: Cardiovascular Imaging*.

[107] Pontone G., Andreini D., Guaricci A. I. (2016). Rationale and design of the PERFECTION (comparison between stress cardiac computed tomography PERfusion versus Fractional flow rEserve measured by Computed Tomography angiography In the evaluation of suspected cOroNary artery disease) prospective study. *Journal of Cardiovascular Computed Tomography*.

[108] Sharma R. K., Arbab-Zadeh A., Kishi S. (2015). Incremental diagnostic accuracy of computed tomography myocardial perfusion imaging over coronary angiography stratified by pre-test probability of coronary artery disease and severity of coronary artery calcification: The CORE320 study. *International Journal of Cardiology*.

[109] Rief M., Zimmermann E., Stenzel F. (2013). Computed tomography angiography and myocardial computed tomography perfusion in patients with coronary stents: prospective intraindividual comparison with conventional coronary angiography. *Journal of the American College of Cardiology*.

[110] Vliegenthart R., De Cecco C. N., Wichmann J. L. (2016). Dynamic CT myocardial perfusion imaging identifies early perfusion abnormalities in diabetes and hypertension: Insights from a multicenter registry. *Journal of Cardiovascular Computed Tomography*.

[111] Rodés-Cabau J., Gutiérrez M., Courtis J. (2011). Importance of diffuse atherosclerosis in the functional evaluation of coronary stenosis in the proximal-mid segment of a coronary artery by myocardial fractional flow reserve measurements. *American Journal of Cardiology*.

[112] Driessen R. S., Stuijfzand W. J., Raijmakers P. G. (2018). Effect of Plaque Burden and Morphology on Myocardial Blood Flow and Fractional Flow Reserve. *Journal of the American College of Cardiology*.

[113] Ahmadi A., Leipsic J., Øvrehus K. A. (2018). Lesion-Specific and Vessel-Related Determinants of Fractional Flow Reserve Beyond Coronary Artery Stenosis. *JACC: Cardiovascular Imaging*.

[114] Nerlekar N., Ha F. J., Cheshire C. (2018). Computed Tomographic Coronary Angiography–Derived Plaque Characteristics Predict Major Adverse Cardiovascular EventsCLINICAL PERSPECTIVE. *Circulation: Cardiovascular Imaging*.

[115] Ziadi M. C. (2017). Myocardial flow reserve (MFR) with positron emission tomography (PET)/computed tomography (CT): Clinical impact in diagnosis and prognosis. *Cardiovascular Diagnosis and Therapy*.

[116] Pontone G., Rabbat M. G., Guaricci A. I. (2017). Stress computed tomographic perfusion: are we ready for the green light?. *Circulation: Cardiovascular Imaging*.

[117] Ishida M., Kitagawa K., Ichihara T. (2016). Underestimation of myocardial blood flow by dynamic perfusion CT: Explanations by two-compartment model analysis and limited temporal sampling of dynamic CT. *Journal of Cardiovascular Computed Tomography*.

[118] Duncker D. J., Koller A., Merkus D., Canty J. M. (2015). Regulation of coronary blood flow in health and ischemic heart disease. *Progress in Cardiovascular Diseases*.

[119] Kim E. Y., Chung W.-J., Sung Y. M. (2014). Normal range and regional heterogeneity of myocardial perfusion in healthy human myocardium: Assessment on dynamic perfusion CT using 128-slice dual-source CT. *The International Journal of Cardiovascular Imaging*.

